# Notice of the Method of Detecting Arsenic by Means of Copper, Proposed by M. Hugo-Reinsch

**Published:** 1844-02-24

**Authors:** Louis-Victor Adouard

**Affiliations:** Beziers


### NOTICE OF THE METHOD OF DETECTING ARSENIC BY MEANS OF COPPER, PROPOSED BY M. HUGO-REINSCH.

By Louis-Victor Adouard of Beziers.

In the number of the "*Journal de Chimie Medicale*" for January, 1843, M. Hugo-Reinsch proposes a new method for detecting arsenic, which he assures us rivals in sensibility the process of Marsh. To convince myself of this fact, the following experiments were undertaken.

Dr. Guy had employed with success at Beziers, Fowler's arsenical solution in certain eruptions. A smith, aged 30 years, by the advice of his physician, took this solution for several days, commencing with two drops morning and evening, and increasing to twelve.

I requested the smith to send me the urine which passed at night and morning, or after each dose of the arsenical solution. I received in a bottle 1200 grammes of urine, perfectly limpid, and slightly acid.

This I divided into two parts; one part was treated by Orfila's process—viz. incineration with nitrate of potassa. This afforded a solution, which gave by Marsh's process an arsenical ring in a glass tube, and spots of the same nature on porcelain; pure materials being used in the process.

On the other hand, the second part of the urine was treated as proposed by M. Reinsch. The liquid was placed in a porcelain capsule, and reduced by evaporation to one-third; on repose, a considerable deposit was formed. To this liquid 200 grammes of pure hydrochloric acid was added; the deposit was immediately dissolved, and the liquid became of a deep brownish red color. Heating the liquid to ebullition, two plates of copper, perfectly clean, were placed in it, and retained for ten minutes. This being without effect, boiling was continued for half an hour, but with a similar result. The plates withdrawn from solution, washed and dried as directed by M. Reinsch, were not covered with a coating of an *iron gray* color; they were brownish, from a coating of brown oxide. One of the plates introduced into a glass tube, and heated, did not yield the least trace of arsenious acid.

These experiments appear to demonstrate, in opposition to the assertions of M. Reinsch, that this method does not rival in delicacy that of incineration by nitrate of potassa, or probably that of carbonization by sulphuric acid, although loss is sustained in both these latter operations.

M. Reinsch asserts, that the method by copper will detect the millionth of 0.050, equal to the 20,000th of 0.001 of arsenious acid. Many experiments made, in which different quantities of arsenious acid had been added to urine, soup, water, &c. enabled me to perceive very distinctly, on the copper, the *iron gray* color, and to obtain afterwards, in a tube, appreciable quantities of arsenious acid, but only when about six-twentieths of 0.001 of the acid was added to the liquid; or the great diversity of six-twentieths, instead of the twenty-thousandth of a milligramme.

I may add, that the method of M. Reinsch, has the serious inconvenience of introducing a salt of copper into the liquid operated on. To confirm this, it is only necessary to evaporate to dryness the liquor treated by copper and hydrochloric acid; calcine the residue, and dissolve in nitric acid, and a considerable amount of distilled water. A solution will be obtained, which will readily demonstrate the presence of copper, by means of ammonia, clean iron, and ferrocyanide of potassium.—*Am. Journ. Pharm., from Journ. de Chim. Med.*

